# Combining sensor tracking with a GPS-based mobility survey to better measure physical activity in trips: public transport generates walking

**DOI:** 10.1186/s12966-019-0841-2

**Published:** 2019-10-07

**Authors:** Basile Chaix, Tarik Benmarhnia, Yan Kestens, Ruben Brondeel, Camille Perchoux, Philippe Gerber, Dustin T. Duncan

**Affiliations:** 1 Sorbonne Université, INSERM, Institut Pierre Louis d’Epidémiologie et de Santé Publique IPLESP, Nemesis team, Faculté de Médecine Saint-Antoine, 27 rue Chaligny, 75012 Paris, France; 2 Department of Family Medicine and Public Health & Scripps Institution of Oceanography, University of California in San Diego, 9500 Gilman Drive #0725, La Jolla, CA 92093 USA; 3 Department of Social and Preventive Medicine, École de Santé Publique de l’Université de Montréal, Centre de recherche du CHUM, Tour Saint-Antoine, 850 Saint-Denis, S03-280, Montréal, H2X 0A9 Canada; 4 University of Montreal Hospital Research Centre, Tour Saint-Antoine, 850 Saint-Denis, S03-280, Montréal, H2X 0A9 Canada; 5 Luxembourg Institute of Socio-Economic Research, Maison des Sciences Humaines, 11 Porte des Sciences, L-4366 Esch-sur-Alzette, Luxembourg; 6 Spatial Epidemiology Lab, Department of Population Health, School of Medicine, New York University, 180 Madison Avenue, New York, NY 10016 USA

**Keywords:** Accelerometry, Global positioning system, Public transport, Transport, Walking

## Abstract

**Background:**

Policymakers need accurate data to develop efficient interventions to promote transport physical activity. Given the imprecise assessment of physical activity in trips, our aim was to illustrate novel advances in the measurement of walking in trips, including in trips incorporating non-walking modes.

**Methods:**

We used data of 285 participants (RECORD MultiSensor Study, 2013–2015, Paris region) who carried GPS receivers and accelerometers over 7 days and underwent a phone-administered web mobility survey on the basis of algorithm-processed GPS data. With this mobility survey, we decomposed trips into unimodal trip stages with their start/end times, validated information on travel modes, and manually complemented and cleaned GPS tracks. This strategy enabled to quantify walking in trips with different modes with two alternative metrics: distance walked and accelerometry-derived number of steps taken.

**Results:**

Compared with GPS-based mobility survey data, algorithm-only processed GPS data indicated that the median distance covered by participants per day was 25.3 km (rather than 23.4 km); correctly identified transport time vs. time at visited places in 72.7% of time; and correctly identified the transport mode in 67% of time (and only in 55% of time for public transport). The 285 participants provided data for 8983 trips (21,163 segments of observation). Participants spent a median of 7.0% of their total time in trips. The median distance walked per trip was 0.40 km for entirely walked trips and 0.85 km for public transport trips (the median number of accelerometer steps were 425 and 1352 in the corresponding trips). Overall, 33.8% of the total distance walked in trips and 37.3% of the accelerometer steps in trips were accumulated during public transport trips. Residents of the far suburbs cumulated a 1.7 times lower distance walked per day and a 1.6 times lower number of steps during trips per 8 h of wear time than residents of the Paris core city.

**Conclusions:**

Our approach complementing GPS and accelerometer tracking with a GPS-based mobility survey substantially improved transport mode detection. Our findings suggest that promoting public transport use should be one of the cornerstones of policies to promote physical activity.

**Electronic supplementary material:**

The online version of this article (10.1186/s12966-019-0841-2) contains supplementary material, which is available to authorized users.

## Background

The public health community is engaged in the promotion of physical activity [1, 2]. A key strategy is to promote active travel modes such as walking. However, walking is often unrealistic in longer trips. As increasing evidence suggests that public transport promotes walking [3, 4], a complementary strategy is to develop public transport as an alternative to private motorized vehicles.

The assessment of walking and physical activity in trips remains imprecise in studies, especially in trips combining walking with other modes. Previous studies, for example, have reported an increase in daily physical activity on days where public transport was used [5], which is an imprecise quantification that lacks information on the time spent in public transport trips on these days and on the exact related physical activity. Other studies have assessed physical activity in trips that were manually identified from GPS data for a restricted number of trips (e.g., home-school trips [6] or home-work trips [7]), lacking an overall picture of physical activity in trips. A third group of studies have automatically detected trips with algorithms but had no information on travel modes [8]. Finally, some studies automatically detected trips and travel modes with algorithms, but did not confirm the travel mode information with participants, so the resulting information might be unreliable and lack details on travel modes (e.g., two-wheel vs. four-wheel vehicle, or private vs. public transport vehicle). However, it is crucial to derive accurate data on the physical activity in trips with different travel modes, for example to provide policymakers with accurate quantitative evidence on the physical activity benefits of public transport use or as input data for subsequent modeling of the population-level impacts on physical activity of scenarios of mode shift and transport policies [3, 9, 10].

**Fig. 1:**
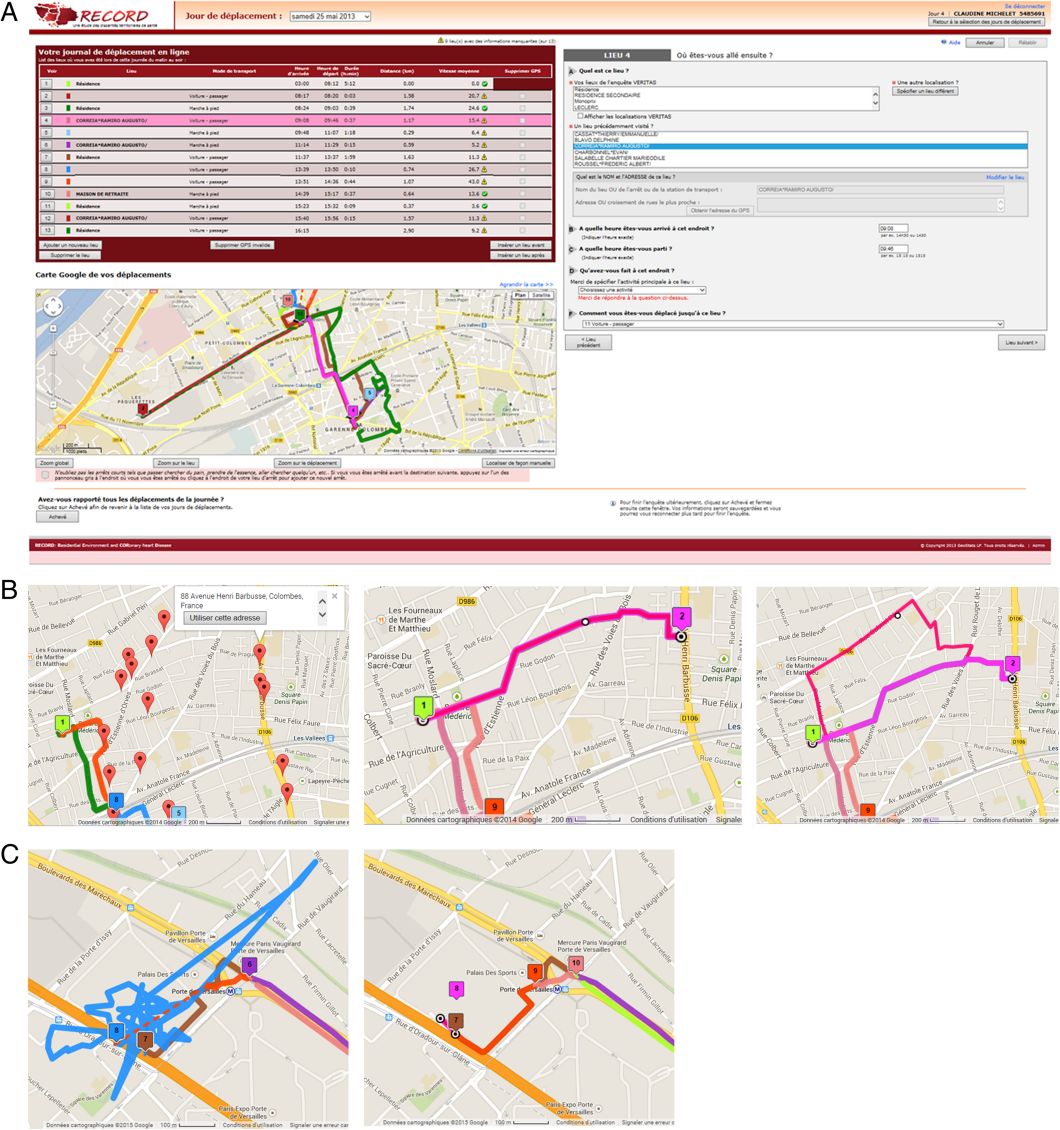
Screenshots of TripBuilder Web used for the GPS-based mobility survey in the RECORD MultiSensor Study. Panel **a**: Main screen of the application (top left: succession of places and points of change of travel mode visited; bottom left: map of trips; right: panel for the identification of places and characterization of places and modes). Panel **b**: A trip was not detected as the GPS receiver was left at home. The visited place was searched in the mobility survey application (first picture). When the adequate place was selected, the shortest trip to the place was generated (second picture). The trip itinerary was then manually edited (third picture). Panel **c**: The residual artefact in the GPS track persisting after the automatic cleaning (first picture) was manually removed during the mobility survey (second picture). The data shown in the Figure are not real participant data but data generated by the research staff

The present work develops novel technologies for the measurement of physical activity in trips. As illustrated in Fig. 1, we propose a novel GPS-based mobility survey strategy, improved compared to our previous work [3, 11], that decomposes trips into trip stages, cleans GPS tracks, and permits the accurate assessment of walking in trips. Table 1 refers to incremental levels of methodology (GPS tracking, accelerometry, mobility survey, decomposition of trips into stages, and full edition of GPS tracks) and indicates the analytical opportunities offered at each level.

**Table 1:** Comparison of our improved measurement of physical activity in trips to existing measurement approaches

Methodology	Added information	Applications
Global and retrospective questionnaire assessment of transport activity (trips, modes)		- Studies of residential characteristics and qualitative outcomes on mode choice
+ GPS data collection & algorithm processing^a^	- Objective and accurate data on trips and places visited (timestamped start and end times of segments, itineraries) instead of self-reported data - Unreliable data on transport modes	- Environmental exposures in daily path areas - Route choice analysis - Analyses focusing on aggregated groups of modes (e.g., motorized transport) and indicators of transport activity aggregated at the participant level (rather than trip-level information)
+ accelerometry & algorithm processing^a^	- Objective information on physical activity in trips	- Participant-level analyses of environmental exposures, transport mode profiles, and physical activity (one observation per participant)
+ GPS-based mobility survey^b^	- Improved accuracy through the confirmation of places visited, trips made, and modes used - Added information on activities at places, social contacts, mood in trips, etc.	- Analyses of environments, transport modes, and physical activity disaggregated at the trip level, with reliable mode information - Individual-level or trip-level analyses of determinants of visiting particular places (a sport facility, a fast-food restaurant, etc.) corrected from the selective daily mobility bias - Analyses involving additional determinants, confounders, or outcomes collected at the trip level
+ spatial/temporal segmentation of trips into trip stages (algorithms + survey)^c^	- Validated information on timestamps and locations for transitions between unimodal trip stages and transfer stages between modes	- Refined analyses of transport mode used accounting for durations spent in the different modes - Analyses of sensor-measured physical activity and personal environmental exposures (noise, air pollutants) by transport modes and during transfer stages between modes - Calculation of exposures to geographic environments by transport modes using geographic information systems
+ manual edition of GPS itineraries (deletion and completion)^c^	- Accurate itineraries associated with each trip stage without artefacts	- Analyses of distances covered with each transport mode - Refined calculation of exposures to geographic environments using geographic information systems

^a^Most studies using GPS and accelerometer data in public health have reached this level

^b^Our previous studies in the field [3, 11] have emphasized the benefits of this approach

^c^The aim of the present paper is to develop these last two improvements in measurement

The methodological aim of this study was to provide information on the accuracy gains offered by this mobility survey, comparing the resulting travel distance and transport mode information to that obtained through the sole algorithm-based processing of GPS data (without mobility survey). Regarding empirical aims, using two alternative metrics (distance walked from GPS and mobility survey and number of steps taken from accelerometry), (i) we compared the amount of walking in trips with different modes, e.g. in entirely walked trips, car trips, and public transport trips; and (ii) we quantified the overall contribution to transport walking of trips with different modes, including public transport.

## Methods

### Data collection and processing

#### Population

Participants came from the RECORD MultiSensor Study [12] of the RECORD Cohort [13–17]. The RECORD MultiSensor Study combined various sensor tools (including a GPS receiver, a waist-worn accelerometer and in subgroups two thigh-worn and chest-worn accelerometers, a cardiac holter, a blood pressure monitor, and a smartphone for ecological momentary assessment) to investigate various aspects of the relationship between transport and health. Participants of the RECORD Cohort were born between 1928 and 1978, were residing at baseline in 10 districts of Paris and 111 other municipalities of the Ile-de-France region, and were recruited without a priori sampling during preventive checkups performed by the IPC Medical Centre.

During the second wave of the RECORD Study, between September 2013 and June 2015, after completing their health checkups, participants were systematically invited to enter the RECORD MultiSensor Study (approved by the French Data Protection Authority) when there were devices available for the recruitment. Of the 919 persons invited to enter the MultiSensor study, 319 accepted to participate and signed an informed consent form. Twenty-seven participants withdrew from the study and the data collection failed for 6 participants, resulting in a final acceptation and completion rate of 31.1% (*N* = 286). Comparison of the RECORD participants who took part in the MultiSensor Study with those who were invited but refused to participate or abandoned showed that the likelihood to participate was twice lower among participants with a primary education or less than among those with an upper tertiary education. One participant who travelled to meet different family members out of the Ile-de-France region during the follow-up was excluded from this analysis (*N* = 285).

#### Collection and processing of GPS, mobility survey, and accelerometer data

Participants wore a QStarz (Taipei, Taiwan) BT-Q1000XT GPS receiver [18] and an Actigraph (Pensacola, FL) wGT3X+ tri-axial accelerometer [19] on the right hip for the recruitment day and 7 additional days. Participants completed a travel diary on the places visited, as supporting information for the mobility survey.

The GPS data (one point every 5 s) were uploaded in the TripBuilder Web mapping application where GPS data were processed with algorithms (Fig. 1) [20, 21]. These algorithms (i) identified the places visited by the participants over 7 days; (ii) decomposed the trips between visited places into segments of trips with unique modes; (iii) imputed information on the activities performed in each place based on the geolocated regular visited places of each participant pre-identified with the VERITAS application [22] and on geolocated points of interest; and (iv) imputed information on the travel mode used in each trip segment based on speeds, survey information on typical modes used by the participant, and on the presence of public transport stations of the same line or mode at the beginning and end of the trip segment.

Based on the TripBuilder Web application, a GPS-based mobility survey was conducted through a telephone interview as soon as possible after the data collection (median time of 10 days, interquartile range: 7, 15). Only the research assistants had access to the application described in Fig. 1, while participants had access to detailed screen copies of their trips sent by postal mail. Using these computer and paper supports, the research assistants walked the participants through the different days, reviewing and complementing information trip by trip. The research assistants confirmed the detected visits to places and trips between these places; they removed visits to places and trips that were incorrect; they could generate visits to places or trips to places undetected by the GPS receiver and/or algorithm (with itineraries then imputed as the shortest street network path and edited if needed, see Fig. 1). The research assistants manually edited each trip itinerary, if needed, to remove residual artefacts in the GPS track that would bias the assessment of the travel distance (Fig. 1). Finally, research assistants confirmed or collected and modified the type of activity practiced at each visited place and the travel mode used in each trip segment.

A SAS program generated a detailed timetable over 7 days indicating the succession of places visited and trips subdivided in trip stages. Within a trip, two trip stages are necessarily separated by an episode of transfer between the two assigned to a punctual location. These transfer episodes coded with a spatial point in the mobility survey typically last from 0 min to several minutes and correspond to no walking at all, walking few meters outdoor, or walking indoor, e.g., within a train or metro station (but these punctual transfer episodes cannot imply movement with any other mode). A transfer between two trip stages by bus would be coded as a walking trip stage if there was a detectable walking track between them, but would be coded as a punctual location if the two buses were few meters apart outdoor. Start/end times are available for each visited place, trip, trip stage, and episode of transfer between trip stages.

Due to costs, the mobility survey was only performed on days (i) where there was GPS data and (ii) where the additional sensors (VitaMove system, Zephyr BioPatch, etc.) employed in this study were worn by the participants. On those days, the mobility survey was systematically performed for the whole day, even if GPS data were partly missing. In the latter case, missing portions of itineraries were complemented during the mobility survey, so that the day had full distance information. On the opposite, if the two conditions above were not satisfied, the whole day was excluded. The study data comprised 1784 days of mobility survey for 285 participants, corresponding to a median of 7 days of follow-up per individual (interdecile range: 4, 7) (i.e., 285 × 7–1784 = 211 days were excluded due to the aforementioned reason).

Choi default parameters applied to vector magnitude data as implemented in ActiLife 6.11.9 were used to identify episodes of nonwear of the accelerometer [23, 24]. Trips that overlapped a nonwear period were flagged. The number of steps was estimated by ActiLife for each epoch.

### Measures

#### Classification of trips

Mobility surveys mostly cover movement between destinations coded as street addresses, but they do not assess movement within the home garden or within an underground transport station. Each trip between two visited places (from a street address to a street address) comprises one or several trip stages (segments of trip with a unique mode). A fully unimodal biking trip is possible if the bike is taken from the departure place to the destination without any walking in the street. A fully unimodal car trip is also possible if parkings are available at the departure place and destination, but if the car is parked in the street, then it would be a multimodal car and walking trip.

Based on the travel mode in each trip stage, a crude and a detailed classifications of trips were defined as follows among trips with a unique mode or with a unique mode in addition to walking. The cruder version of the variable distinguished: entirely walked trips; biking or use of rollers or of a skateboard (“other active modes”); public transport; personal motorized vehicle; and other (long distance train and plane, i.e., non-local trips). A more detailed classification subdivided public transport into: bus/coach; metro (available in Paris and immediate surroundings); RER (fast trains traveling through Paris and the suburbs), train, or TER (trains from Paris towards suburbs or adjacent regions) (referred to below as suburban trains); and tramway. Personal motorized vehicle was subdivided into driving a personal motorized vehicle and being a passenger of a personal motorized vehicle (including taxi). In either the crude or the detailed classification, trips that comprised two stages or more with different non-walking modes (as defined in the corresponding classification) were labeled as multi-mode trips.

#### Distance walked

We were able to calculate accurate walked and nonwalked distances because the GPS tracks were carefully edited and cleaned and because missing trips or trip segments were recreated during the phone mobility survey in our web mapping application. The walked or nonwalked distance covered in each trip stage was the length of the corresponding polyline. By definition, there is no distance related to the episodes of change of mode within trips, as they are represented as point locations. For calculation purposes, we also aggregated the walked and nonwalked distances at the trip level.

A first definition of the intensity of walking in a walked segment was the average speed of walking in km/h.

#### Accelerometer-assessed steps

The accelerometer-assessed number of steps taken was aggregated for each trip stage and also for episodes of transfer between trip stages, according to the start/end times of each segment.

A second definition of the intensity of walking (in walked trips or trip stages) was the number of accelerometer steps taken per min.

#### Sociodemographic and geographic covariates

Age was used as a continuous variable. Education was coded in 4 categories: no education, primary education, or lower secondary education; higher secondary education and lower tertiary education; intermediate tertiary education; and upper tertiary education. Employment status was categorized in 4 classes: stable job; unstable and precarious job; unemployed; and other. The urbanicity degree of the area of residence was assessed with a 3-category variable: Paris; close suburb (first circle of counties adjacent to Paris); and far suburb (second circle of counties non-adjacent to Paris). Two participants who were in an alternative residence all over the observation period were assigned to the geographic location of this alternative residence within the Ile-de-France region.

### Statistical analysis

#### Analytical sample

The initial timetable for 285 participants comprised 31,115 segments of observation (either trip stages, episodes of transfer of mode, or places visited), corresponding to 9046 trips and 9369 (non-unique) places visited. We excluded from this timetable the episodes at the places visited, yielding a sample of trips (21,354 segments of observation, either trip stages or episodes of transfer).

The analyses excluded certain trip stages or segments of trips corresponding to non-trip movement. First, for sequences of movement over space between two places visited that included a reported segment by skiing or chairlift, the whole sequence of movement was deleted [*n* = 95 segments of observations (trip stages or episodes of transfer) corresponding to 5 trips]. Second, for sequences of movement over space that included a reported segment of jogging or walking a dog, only the corresponding segment was excluded together with the eventually preceding or subsequent short episodes of transfer (change of mode) (*n* = 96 segments of observation), but trip stages with other modes within the trip were not deleted (e.g., driving or walking before or after the jogging episode itself). We did not exclude non-local trip stages with long distance trains (*n* = 15 trip stages) or planes (*n* = 4 trip stages), as they pertain to transport contrary to skiing, jogging, or walking a dog. The final sample comprised 8983 trips, corresponding to 21,163 segments of observation.

To analyze the accelerometer-derived number of steps, we further excluded a participant for whom the accelerometry follow-up did not work (*n* = 34 trips) and trips that overlapped a period of nonwear of the accelerometer (*n* = 221) (exclusion was made at the trip level even if there was nonwear only in some stages of the trip). The sample for analyzing accelerometry comprised 284 participants with 8728 trips corresponding to 20,564 segments of observation.

#### Quantification of accuracy gains from the mobility survey

To compare our approach combining GPS tracking and a mobility survey with the simpler approach only relying on GPS tracking and processing algorithms, first, we compared the estimated total distance covered per day by participants over the follow-up, as assessed in 3 different ways: (i) with the almost raw GPS data [i.e., the GPS data after the sole exclusion of GPS points that were below the speed limit of 1 km/h, as specified in the published algorithm on which we rely [20, 21]; (ii) with the trip distances calculated after the identification of places visited and trips between them through automatic algorithms [20, 21]; and (iii) with the trip distances determined after the manual edition and complementation of GPS tracks through the mobility survey.

Second, we compared data on transport modes automatically identified with algorithms from the GPS data [20, 21] to the transport mode data on the same set of trips derived from the full GPS-based mobility survey (i.e., eventually corrected during the survey). We report the percentage of time over which the two sources of mode information agree, overall and by transport modes.

#### Analysis of walking distance and number of steps taken

Sociodemographic characteristics were only used for the description of the sample. The distance walked and the accelerometer-assessed number of steps per trip were tabulated according to the main travel mode in the trip (crude and detailed classifications). Differences according to the mode used were tested with the Kruskal-Wallis test. We calculated the percentage of the overall distance walked and accelerometer-assessed steps that were covered in each type of trip according to the crude and detailed classification of modes at the trip level.

## Results

### Quantification of accuracy gains from the mobility survey

From the quasi-raw GPS data, the median distance covered by participant per day was of 39.3 km (interdecile range: 16.9, 98.4 km). From the algorithm-based trip distances (uncorrected through the mobility survey), this median distance was of 25.3 km (interdecile range: 8.7, 78.2 km). Finally, when considering the GPS-based mobility survey data, the distance covered by participant per day was reduced to a median of 23.4 km (interdecile range: 7.5, 77.6 km).

Of the time spent in transport as assessed from the GPS-based mobility survey, 72.7% was also identified as corresponding to transport with the algorithm (and reciprocally, 75.7% of the algorithm-based transport time was confirmed as transport time through the mobility survey). Among segments identified as transport with both the algorithm and the mobility survey (over a cumulated period of 86 days, 3 h and 59 min), the transport mode was correctly assessed by the algorithm processing of GPS data for 67% of the time (compared to the GPS-based mobility survey as our gold standard). When stratified by transport mode, the mode was correctly identified by the algorithm for 68% of time for personal motorized vehicle, for 55% of time for public transport (i.e., 63% for the bus/coach, 69% for the metro, 45% for suburban trains, and 22% for the tramway), and for 88% of the time for walking.

### Descriptive data on participants and trips

In the sample of 285 participants, mean age was 50.2 (interdecile range: 37, 63). Sixty-three percent of participants were males; 73% had a permanent job, 3% a temporary job, and 4% were unemployed; 53% had 3 or more years of University education; 35% of the participants were living in Paris, 45% in the close suburb, and 20% in the far suburb.

Considering observation periods covered by the mobility survey, these participants spent a median of 7.0% of their total time including sleep time in trips (transport activity) (interdecile range among 285 participants: 3.6, 11.4%). The number of trips per day per participant (excluding jogging, walking a dog, or skiing segments) had a median of 5 (interdecile range: 3, 7), corresponding to 8 trips stages per individual per day (interdecile range: 4, 13). In the distribution of trips, there were 1.75% (*n* = 157) of multi-mode trips (several modes in addition to walking) according to the crude classification, and 6.96% (*n* = 625) according to the detailed classification of modes. Among trips with a unique mode (or involving a unique mode in addition to walking), 42.3% of trips were entirely walked trips; 4.8% were biking/rollers/skateboard trips; 3.0% of trips were with buses/coaches, 6.2% with metros, 1.8% with suburban trains, and 0.8% with tramways; and 36.0 and 5.0% of trips relied on a personal motorized vehicle as the driver or passenger, respectively.

### Distance walked

Participants covered a median (walked and nonwalked) distance of 22.1 km per day in trips (interdecile range: 7.1 km, 77.6 km). They walked a median of 2.1 km per day over all types of trips (interdecile range: 0.6 km, 4.5 km). As shown in Table 2, the median distance walked per trip was of 0.40 km for entirely walked trips, while it was of 0.85 km for public transport trips. As expected, the median distance walked was almost twice for trips including a stage with a surburban train (1.20 km) than for trips with metros or buses that are often spatially accessible on a more local basis. Overall, 54.1% of the total distance walked in trips was covered in entirely walked trips, while as much as 33.8% of this total distance walked was accumulated during public transport trips, as compared to 8.3% in trips with a personal motorized vehicle.

**Table 2:** Overall distance and distance walked in trips according to the main mode used in the trip

Classifications of trips according to the mode	Distance walked per trip in km: median (10th and 90th percentiles)	Cumulated distance walked per individual^a^ per day in km: median (10th and 90th percentiles)	Cumulated (walked and non-walked) distance per individual^a^ per day in km: median (10th and 90th percentiles)	% of distance walked attributable to these trips
Crude classification				
Entirely walked trips	0.40 (0.11, 1.25)	0.94 (0.18, 2.67)	0.94 (0.18, 2.67)	54.1
Other active modes	0.00 (0.00, 0.39)	0.00 (0.00, 0.00)	0.00 (0.00, 1.51)	1.1
Public transport	0.85 (0.36, 1.61)	0.50 (0.00, 2.22)	3.63 (0.00, 20.56)	33.8
Private motorized	0.00 (0.00, 0.31)	0.07 (0.00, 0.52)	9.06 (0.00, 57.18)	8.3
Other^b^	0.09 (0.00, 0.17)	0.00 (0.00, 0.00)	0.00 (0.00, 0.00)	0.0
Multi-mode	0.59 (0.09, 1.26)	0.00 (0.00, 0.19)	0.00 (0.00, 7.52)	2.7
Detailed classification				
Entirely walked trips	0.40 (0.11, 1.25)	0.94 (0.18, 2.67)	0.94 (0.18, 2.67)	54.1
Other active modes	0.00 (0.00, 0.39)	0.00 (0.00, 0.00)	0.00 (0.00, 1.51)	1.1
Bus/coach	0.63 (0.00, 1.34)	0.00 (0.00, 0.31)	0.00 (0.00, 1.69)	4.4
Metro	0.79 (0.39, 1.39)	0.00 (0.00, 1.03)	0.00 (0.00, 6.43)	11.1
Suburban train	1.20 (0.63, 1.99)	0.00 (0.00, 0.41)	0.00 (0.00, 2.26)	4.9
Tramway	1.11 (0.52, 1.68)	0.00 (0.00, 0.00)	0.00 (0.00, 0.00)	1.9
Private motorized (driver)	0.00 (0.00, 0.28)	0.00 (0.00, 0.45)	5.55 (0.00, 50.32)	6.2
Private motorized (passenger)	0.00 (0.00, 0.50)	0.00 (0.00, 0.09)	0.00 (0.00, 8.31)	2.0
Other^b^	0.09 (0.00, 0.17)	0.00 (0.00, 0.00)	0.00 (0.00, 0.00)	0.0
Multi-mode	0.84 (0.30, 1.59)	0.00 (0.00, 1.20)	0.22 (0.00, 23.95)	14.4

RECORD MultiSensor Study, 285 participants, 8983 trips, 21,163 trip stages

^a^In the second and third columns, distances are drawn from the distribution of cumulated distances over the 285 individuals, including those who do not use the corresponding modes

^b^Long distance train and plane

Regarding geographic disparities, participants accumulated a median distance walked per day in all trips of 2.6 km in Paris, of 2.0 km in the close suburb, and of 1.5 km in the far suburb. As shown in Table 3 (top part), the percentage of the total distance walked in trips accumulated in entirely walked trips was fairly comparable in Paris and in the close and far suburbs. However, the percentage of the total distance walked in trips that was covered in public transport trips decreased by 10 percentage points from Paris to the far suburb while the percentage that was accumulated in trips with a personal motorized vehicle symmetrically increased by 10 percentage points.

**Table 3:** Percentages of distance walked and percentages of steps taken^a^ attributable to trips with different modes

	Paris	Close suburb	Far suburb
% of distance walked			
Entirely walked trips	54.4	53.6	54.3
Other active modes	2.0	0.5	0.1
Public transport	36.9	33.3	26.8
Private motorized	4.4	10.0	14.4
Other^b^	0.0	0.0	0.0
Multi-mode	2.3	2.6	4.4
% of steps taken			
Entirely walked trips	45.5	41.9	40.6
Other active modes	4.5	2.9	1.2
Public transport	39.7	38.4	28.1
Private motorized	5.6	13.5	23.8
Other^b^	0.0	0.0	0.0
Multi-mode	4.7	3.4	6.4

Percentages tabulated for participants residing in different geographic environments (RECORD MultiSensor Study)

^a^We consider the steps accumulated over the entire trip (including with non-walking modes and during transfer episodes)

^b^Long distance train and plane

As shown in Table 4 (first column), the recorded speed of walking in km/h was higher in the walking stages of trips with public transport than in entirely walked trips (Kruskal-Wallis test, *p* < 0.0001).

**Table 4:** Speed of walking and number of steps taken per minute

Type of trips or trip stages	Speed of walking^a^ in km/h: median (10th and 90th percentiles)	Number of steps taken per minute^b^: median (10th and 90th percentiles)
Entirely walked trips	5.0 (2.9, 7.8)	83.5 (7.8, 112.7)
Walking trip stages of public transport trips		
All public transport trips	5.4 (3.2, 8.6)	84.1 (11.0, 115.0)
Bus/coach trips	5.4 (3.0, 10.1)	81.3 (17.2, 115.1)
Metro trips	5.3 (3.1, 8.3)	82.1 (11.0, 113.3)
Suburban train trips	5.3 (3.2, 7.5)	94.8 (32.9, 115.1)
Tramway trips	5.8 (4.0, 7.8)	89.7 (8.3, 116.0)

Indicators of the intensity of walking are provided for walked trips and for walking stages of public transport trips (RECORD MultiSensor Study)

^a^In this calculation, the trips and trip stages were weighted according to the distance walked

^b^In this calculation, the trips and trip stages were weighted according to the duration walked

### Accelerometer-derived steps

Over the mobility survey period, the accelerometer wear time per day had a median value of 14hr02min across the 284 participants (interdecile range: 10hr37min, 15hr48min). Participants accumulated a median of 4280 steps per unit of 8 h of accelerometer wear time (interdecile range: 2670 steps, 6842 steps). Over the entire accelerometer wear time, 39.6% of steps taken (interdecile range: 16.5, 59.6%) were accumulated during trips as opposed to visits at places.

As shown in Table 5, the number of steps taken per trip had a median value of 425 in entirely walked trips, while it was of 1352 in public transport trips. Again, trips relying on suburban trains were associated with a higher number of steps taken per trip (median = 1933) than trips with buses or metros. Overall, 43.2% of all steps taken during trips were covered in entirely walked trips, while 37.3% of such steps were covered in public transport trips, as compared to 11.7% in trips with a personal motorized vehicle.

**Table 5:** Accelerometer-derived number of steps taken in trips according to the main mode^a^ used in the trip

Classifications of trips according to the mode	Number of steps taken per trip: median (10th and 90th percentiles)	Cumulated number of steps in trips per individual per 8 h of accelerometer wear time:^b^ median (10th and 90th percentiles)	% of trip-related steps taken attributable to these trips
Crude classification			
Entirely walked trips	425 (4, 1587)	554 (61, 2176)	43.2
Other active modes	283 (41, 1007)	0 (0, 123)	3.3
Public transport	1352 (499, 2453)	397 (0, 1913)	37.3
Private motorized	86 (10, 453)	120 (0, 549)	11.7
Other^c^	14 (2, 26)	0 (0, 0)	0.0
Multi-mode	1213 (445, 2402)	0 (0, 234)	4.4
Detailed classification			
Entirely walked trips	425 (4, 1587)	554 (61, 2176)	43.2
Other active modes	283 (41, 1007)	0 (0, 123)	3.3
Bus/coach	771 (169, 1910)	0 (0, 243)	4.3
Metro	1267 (576, 2292)	0 (0, 892)	12.6
Suburban train	1933 (859, 2876)	0 (0, 265)	5.4
Tramway	1286 (271, 1957)	0 (0, 0)	1.5
Private motorized (driver)	83 (10, 420)	72 (0, 476)	9.4
Private motorized (passenger)	118 (12, 705)	0 (0, 105)	2.2
Other^c^	14 (2, 26)	0 (0, 0)	0.0
Multi-mode	1466 (638, 2480)	0 (0, 1165)	18.1

RECORD MultiSensor Study, 284 participants, 8728 trips, 20,564 trip stages

^a^We consider the steps accumulated over the entire trip (including with non-walking modes and during transfer episodes)

^b^The overall number of days of mobility survey could not be used as the denominator as there is nonwear of the accelerometer within it. As a consequence, we chose as the denominator the number of units of 8 h of accelerometer wear time

^c^Long distance train and plane

Regarding geographic disparities, participants accumulated in all trips a median number of steps per 8 h of accelerometer wear time of 1994 in Paris, of 1442 in the close suburb, and of 1275 in the far suburb. Slightly differently than for the geographic disparities in distance walked, the percentage of trip-related accelerometer steps that were accumulated in entirely walked trips slightly decreased from Paris to the far suburb (Table 3, bottom part). While the percentage of trip-related steps taken in public transport trips decreased by 10 percentage points from Paris to the far suburb, the percentage of steps accumulated in trips with a personal motorized vehicle increased from 5.6% in Paris to 23.8% in the far suburb.

As reported in Table 4 (second column), although the figures were close to each other, the recorded number of steps per minute was higher in walking trip stages of public transport trips than in entirely walked trips (Kruskal-Wallis test, *p* = 0.003).

## Discussion

The present study developed novel methodologies for jointly collecting and processing GPS, mobility survey, and accelerometer data that enable an accurate assessment of physical activity in trips.

### Strengths and limitations of the approach

The key novelty of the present study is its innovative GPS-based methodology involving a strong algorithmic pre-processing of GPS data, a phone-administered mobility survey, and a detailed manual correction and complementation of GPS tracks, allowing the timestamping and geolocation of each trip start and end points and points of change of mode within trips. To the best of our knowledge, the RECORD MultiSensor Study is the first to apply this comprehensive methodology in public health research, and the first to combine it with accelerometers and other sensors [12]. As recently reviewed [25], an alternative strategy is to ask study participants to report information on their trips in a paper or electronic diary [26], and to a posteriori link these travel mode data to trips identified from GPS data [7, 27]. However, the real-time reporting of information on activities and modes is burdensome and leads to high rates of missing data [28]. Moreover, a challenge is that the two separately collected sources of data on the same trips, the GPS data and the diary data, then must be aligned using imperfect decision rules, which implies approximations [5, 26].

The proposed GPS-based prompted recall mobility survey approach, rooted in transport sciences [20, 29–31], aims to address these two concerns. First, our participants were asked to fill a simple travel diary. However, this is not our only source of information on travel modes, but just a complementary tool to support the recall during the phone mobility survey, so missing data in this diary are less critical. Moreover, advanced algorithms aim to automatically identify visited places (on the basis of previously surveyed regular destinations and points of interest) and travel modes (on the basis of speeds, usual modes reported in a survey, and geographic location of public transport stations). This pre-identification is important to reduce the burden of the survey for both the participants and research assistants (who confirm the detected modes in each trip based on the diary and with the participant on the phone). Second, there is no need of imprecise a posteriori alignment of GPS trips and survey travel modes as in the alternative approach, since the travel modes are pre-identified and then confirmed / collected during the phone mobility survey on the basis of GPS trips.

Compared to the mobility survey that we implemented in our previous RECORD GPS Study [3, 9, 11, 32], in this new RECORD MultiSensor Study based on a novel GPS-based web survey application, we now perform a full and accurate correction and complementation of GPS tracks. Even after removing unreliable GPS points (e.g., with an excessive dilution of precision or excessive speed), numerous artefacts remain in the GPS tracks that completely preclude the reliable calculation of distances covered from GPS data, an important information from a transport and public health perspective. We therefore carefully edited all the GPS tracks, removing any artefact in the tracks and complementing the tracks when needed, e.g., when short segments of walk before or after public transport episodes were missing. It enabled an accurate assessment of trip distances and walked distances, including within trips with heavier modes.

Our approach has its own limitations. Despite an improved precision, the timestamping of starts and ends of trips and of changes of modes within trips can lack accuracy, especially when GPS data are lacking and when research assistants then have to approximately assign timestamps. It could affect the assessment of accelerometer physical activity in short walking episodes, e.g., in indoor transfer walks between public transport stages.

Transfer walks from one to another rail-based trip stages often occur underground in the Paris region. Most instances of these transfers are coded in our mobility survey as point locations connecting two rail-based itineraries. While there is obviously no map distance associated with these transfer episodes in our survey, they have start and end times, and it is possible to calculate statistics on the number of accelerometer steps per minute during these very short transfer episodes. However, because the timestamps of these underground transfer episodes lack accuracy, the corresponding statistics are reported in Additional file 1: Table S1. It is not clear whether the lower number of steps per minute in these transfer episodes than in other walked segments is attributable to the expected waiting times or also to imprecise timestamping.

Clearly, another limitation of the proposed approach is its cost. Depending on the data collected in the mobility survey, correcting and complementing 7 days of a participant’s data can take one full day of work for a research assistant. Trained research assistants able to apply a large number of coding and processing rules are needed. However, we emphasize below and in Table 1 that GPS-based mobility surveys allow one to collect specific data offering analytical opportunities that would not be available otherwise. And spending substantial amounts of money per participant to collect high quality data is common in research (e.g., for assessing genetic variants or biomarkers), so there is no reason why researchers should not similarly invest to collect reliable data on transport behavior, time budgets, places visited, and activities. Moreover, Table 1 shows that less time-consuming mobility surveys collecting a lower amount of data or with a lower accuracy than the one implemented here are also possible, depending on the research aims and analytical capabilities needed (e.g., collecting timestamps for trips but not for trip stages, or collecting detailed timestamps without manual editing of GPS tracks).

### Accuracy gains from the mobility survey

When participants are at a fixed location (e.g., in a building) and the GPS receiver keeps logging data (through the windows), it often generates pseudo-ambulations over considerable (fake) distances. The substantial drop in the distance covered by participants when comparing the quasi-raw GPS data to the algorithm-processed data is attributable to the elimination of a large share of these pseudo-ambulations. Similarly, the additional although smaller drop in the total distance covered in trips from the algorithm-based to the mobility survey-based versions of the statistic was due to the manual elimination of residual pseudo-ambulations. This second drop in distance underestimates the extent of the manual correction, as on the other hand we also added trips that were missed by the GPS receiver.

In addition to an inadequate assessment of distances covered, the algorithm-processed GPS data (uncorrected through the mobility survey) were substantially mistaken in their distinction of transport time from time spent at a visited place; and they were also massively wrong in their identification of transport modes, and particularly so for public transport. Thus, our study reveals that it would be unwise to investigate the relationship between transport modes in trips and the corresponding physical activity, e.g., assessed with accelerometers, using these GPS-based algorithm-identified transport mode data.

### Interpretation of empirical findings

Our method enabled to quantify physical activity in trips using two accurate metrics that provided coherent findings. For example, public transport trips were associated with a more than twice larger median distance walked per trip, and with a three times larger median number of steps taken per trip, as compared to entirely walked trips. Also, the two metrics (distance walked and number of steps) indicated that, among public transport trips, trips involving a suburban train implied the larger walking activity per trip while those involving buses implied the lower walking activity. This observation is attributable to the typically shorter distance to reach bus stops than suburban train stations.

Over the observation period, 33.8% of the total distance walked during trips and 37.3% of all steps taken in trips were attributable to public transport trips. While these two figures are clearly coherent, the higher percentage attributable to public transport for the steps taken than for the distance walked is likely due to the fact that the metric of accelerometer steps also captures walking within transport stations and underground settings, including during transfer episodes coded as punctual locations in terms of spatial distance. These figures, as the main empirical finding of the paper, suggest that public transport is a major generator of physical activity. Promoting public transport use for trips that are difficult or impossible to walk or bike should be one of the cornerstones of public policies to promote physical activity.

Our analysis of geographic disparities showed that residents of the far suburbs cumulated a 1.7 times lower distance walked per day during trips and 1.6 times fewer trip-related steps per 8 h of accelerometer wear time than residents of Paris. This is attributable to the sharply lower reliance on the two main sources of transport physical activity in the far suburbs, where the number of walking trips per individual per day was more than two times lower and the number of public transport trips per individual per day three times lower than in Paris (as shown in Additional file 1: Table S2). As a result, public transport trips had a lower contribution and trips with a personal motorized vehicle a higher contribution to the lower amount of transport-related walking in the far suburb than in Paris. These findings strongly support the idea that further developing public transport in the close and far suburbs of Paris would reduce geographic disparities in transport-related physical activity.

Finally, another illustration of the accuracy of our methodology is that the two metrics of the intensity of walking (speed of walking and number of steps per minute) coherently indicated a higher intensity of walking during the walking stages of public transport trips than during entirely walked trips. An obvious reason is the inflexible constraint related to the departure time of public transport vehicles in the former.

### The importance of mobility survey data

Table 1 summarizes the analytical opportunities offered by GPS-based mobility surveys, describing the benefits associated with each additional layer of refinements introduced in the survey. We discuss here the benefits of a GPS-based mobility survey against the simpler strategy involving only sensor-based tracking.

First, as illustrated in this paper, our reliance on a mobility survey in addition to the sensor-based data collection allowed us to provide accurate figures on the transport behavior and transport-related physical activity of participants that other methodologies such as the sole processing of sensor data by algorithms could not provide. Such accurate figures are needed for the correct information of policymakers and, as illustrated in previous articles [3, 9, 10], as input data for modeling the impact of various scenarios of interventions using simulation work. In this previous work, we modelled physical activity in trips in function of trip characteristics with random forests techniques in our small sensor-based sample, and then we applied this random forest algorithm to predict physical activity in each trip of participants from a large representative transport survey, and finally used this large transport survey sample to assess through simulations the impact of scenarios of shift in transport modes (public policies) on population physical activity [9, 10]. Such a work would not have been possible with a reasonable degree of accuracy without our GPS-based mobility survey. As detailed in Table 1, the quality of study findings on transport activity will vary depending on the precision of the mobility survey (i.e., whether segmentation into trip stages and manual edition/complementation of GPS tracks is conducted or not).

Second, such GPS-based mobility survey data provide a background of timestamped information on activities and travel modes against which to interpret the data collected with other sensors. For example, similar to what we did with accelerometry, when mobility survey data are combined with sound pressure and air pollutant data collected with wearable monitors, it is possible to isolate transport-related or even mode-specific exposures. Similarly, such mobility survey information can be used to calculate transport- or mode-specific built environmental exposures along GPS tracks with a geographic information system.

Third, a mobility survey provides a tool and opportunity for collecting other information, either on the exposure side or on the outcome side, disaggregated over space and time. For example, we used or are using such a mobility survey to collect data on recreational physical activity in trips and at visited places in our RECORD MultiSensor Study, on social contacts in our RECORD-HANC protocol, and on stress in trips in our MobiliSense protocol. Such spatially and temporally disaggregated data are needed to develop life-segment and momentary analyses of relationships, as recommended elsewhere [25].

Fourth, collecting such data on activities at visited places is critical to address the selective daily mobility bias discussed in previous articles [22, 25, 33]. For example, it is useful to collect information on places specifically visited to practice sports or to purchase or consume foods, because such places should not be considered as reference locations when calculating the spatial accessibility to facilities in studies investigating accessibility effects on the corresponding behavior.

## Conclusions

Combining a GPS and accelerometer data collection with a GPS-based mobility survey allowed us to explore the walking physical activity in transport using two complementary metrics, i.e., distance walked and accelerometer steps. Methodologically, our comparison of GPS-based mobility survey data with algorithm-only processed GPS data suggests that the latter substantially distort transport modes, and thus would yield biased findings. Empirically, our results, which might only apply to cities within a comparable urban structure and transport infrastructure, suggest that public transport is a major generator of physical activity, with a steady decrease from the core of Paris to the far suburb in the overall transport-related walking activity and in the contribution of public transport to walking activity.

## Additional file

Additional file 1: **Table S1.** Number of steps taken per minute during episodes of transfer in public transport trips represented as point locations in the mobility survey (RECORD MultiSensor Study). **Table S2.** Average number of trips per individual per day according to the main mode in the trip and according to the geographic location of the residence (RECORD MultiSensor Study). (DOCX 17 kb)

## Data Availability

The dataset analyzed in the current study is not publicly available due to confidentiality of mobility destinations of participants but may be available from the corresponding author on request.
