# Supplementary material for: Combining sensor tracking with a GPS-based mobility survey to better measure physical activity in trips: public transport generates walking
Source: Int J Behav Nutr Phys Act. 2019 Oct 7;16:84. doi: 10.1186/s12966-019-0841-2 (PMC6781383; doi:10.1186/s12966-019-0841-2)
Supplement: Supplementary file 1 — Table S1. Number of steps taken per minute during episodes of transfer in public transport trips represented as point locations in the mobility survey (RECORD MultiSensor Study). Table S2. Average number of trips per individual per day according to the main mode in the trip and according to the geographic location of the residence (RECORD MultiSensor Study). (DOCX 17 kb) [file 12966_2019_841_MOESM1_ESM.docx]

**Additional file 1**

| **Table S1.** Number of steps taken per minute during episodes of transfer in public transport trips represented as point locations in the mobility survey (RECORD MultiSensor Study) | |
| --- | --- |
|  | Median (10^th^ and 90^th^ percentiles)^a^ |
| Points of change of modes in public transport trips |  |
| All public transport trips | 16 (1, 80) |
| Bus/coach trips | 11 (1, 57) |
| Metro trips | 22 (0,101) |
| Suburban train trips | 18 (2, 72) |
| Tramway trips | 13 (0, 68) |
| ^a^The episodes of transfer were weighted according to their duration. | |

| Table S2**.** Average number of trips per individual per day according to the main mode in the trip and according to the geographic location of the residence (RECORD MultiSensor Study) | | | |
| --- | --- | --- | --- |
| Classifications of trips according to the mode | Paris | First crown of counties | Second crown of counties |
| Walking only | 2.8 | 1.8 | 1.3 |
| Other active modes | 0.3 | 0.2 | 0.1 |
| Public transport | 1.2 | 0.8 | 0.4 |
| Private motorized | 1.0 | 2.1 | 3.0 |
| Other^a^ | 0.0 | 0.0 | 0.0 |
| Multi-mode | 0.1 | 0.1 | 0.1 |
| ^a^Long distance train and plane | | | |
